# A Rare Case of Kikuchi-Fujimoto Disease

**DOI:** 10.7759/cureus.39098

**Published:** 2023-05-16

**Authors:** Abeer Qasim, Vikram Itare, Muhammad Yasir Anwar, Esther Arguello Perez

**Affiliations:** 1 Internal Medicine, BronxCare Health System, Bronx, USA; 2 Infectious Disease, BronxCare Health System, Bronx, USA

**Keywords:** kikuchi-fujimoto disease (kfd), autoimmune disease, syndrome of fever of unknown origin, necrotizing lymphadenitis, kikuchi disease

## Abstract

Kikuchi-Fujimoto disease (KFD) is an autoimmune condition that is more common in females and occurs in the third decade of life. The condition is usually benign and self-resolving and is characterized by fever, cervical lymphadenopathy, night sweats, myalgia, and rashes. The disease can be misdiagnosed as reactive follicular hyperplasia, tuberculous lymphadenitis, systemic lupus erythematosus, and malignant lymphoma. The diagnosis of KFD involves the excision of the affected lymph node. Although there is no specific treatment for the disease, usually symptomatic and supportive measures are effective; however, steroids and immunosuppressive therapies are considered in more severe cases. The disease lasts for around one to four months. The neurological complications include cerebellar ataxia, meningoencephalitis, and aseptic meningitis. Here, we describe the case of a 36-year-old male who presented with complaints of fever, malaise chills, anorexia, and fatigue associated with a tender right axillary lymph node. The patient underwent a biopsy which confirmed KFD and responded well to supportive therapy.

## Introduction

Kikuchi-Fujimoto disease (KFD), also known as histiocytic necrotizing lymphadenitis, is a rare and benign disorder primarily affecting young adults [1]. The disease is characterized by tender, swollen lymph nodes associated with fever, generalized malaise, and night sweats. The exact cause of KFD remains unknown. However, theories have suggested that it is an autoimmune response to a viral infection or caused by other environmental triggers [2]. The diagnosis of KFD is based on the clinical course and histopathology of the affected lymph node. The treatment is mainly symptomatic, including antipyretics and corticosteroids [3].

This article aims to report a case of KFD which can be considered in the differential diagnosis of fever of unknown origin or prolonged fever, especially with lymphadenopathy and not responding to broad-spectrum antibiotics.

## Case presentation

A 36-year-old male with a known history of syphilis, human immunodeficiency virus 2, and chronic hepatitis B presented to the Emergency Department in August complaining of fever, chills, anorexia, fatigue, and generalized body aches for one day before the presentation. The patient denied recent travel history and sick contacts. In addition, the patient reported having two cats at home. In the Emergency Department, the patient was febrile with a temperature of 102.5°F, a pulse of 105 beats per minute, a respiratory rate of 18 breaths per minute, a blood pressure of 117/77 mmHg, and normal oxygen saturation of 99% in room air.

The physical examination was unremarkable. His initial laboratory testing revealed mild leukopenia (white blood cell count of 4.3k/µL), increased D-dimer of 422 ng/mL, and acute kidney injury, with a creatinine level of 1.6 mg/dL, increased from a baseline of 1.1 mg/dL (Table 1).

**Table 1:** Initial laboratory results.

Laboratory parameters	Results	Reference ranges and units
Red blood cell count	5	4.50–5.90 million/µL
Hemoglobin	15.1	12.0–16.0 g/dL
Hematocrit	43.6	42–51%
Platelet	217	150–400 k/µL
D-dimer assay, plasma	422	0–230 ng/mL
Sodium, serum	137	135–145 mEq/L
Potassium, serum	4	3.5–5.0 mEq/L
Blood urea nitrogen, serum	16	8–26 mg/dL
Creatinine, serum	1.6	0.5–1.5 mg/dL
Bilirubin, serum total	0.4	0.2–1.1 mg/dL
Bilirubin, serum direct-conjugated	<0.2	0.0–0.3 mg/dL
Alkaline phosphatase, serum	60	56–155 U/L
Aspartate transaminase, serum	36	9–48 U/L
Alanine aminotransferase, serum	17	5–40 U/L
Lactic acid level	1.0	0.5–1.6 mmol/L
Lactate dehydrogenase, serum	221	110–210 U/L
Haptoglobin, serum	336	30–200 mg/dL
Ferritin	153	13–150 ng/mL
C-reactive protein	4.01	<5 mg/dL
Urine toxicology	Amphetamine positive	
HIV RNA quantitative polymerase chain reaction	Non-detectable	
Hepatitis B virus DNA copies/mL	Non-detectable	

Intravenous fluids and broad-spectrum antibiotics (vancomycin and piperacillin/tazobactam) were administered to the patient after collecting two blood and urine cultures. The respiratory panel polymerase chain reaction (PCR) for adenovirus, rhinovirus/enterovirus, influenza A subtypes H1 and H3, influenza B, human metapneumovirus, human respiratory syncytial virus A/B, and human parainfluenza virus 1, 2, and 3 was negative. In addition, PCR for SARS-CoV-2 was negative. After three days of a persistent fever (Table 2), despite broad-spectrum antibiotics and negative blood and urine cultures, a computed tomography (CT) scan of the chest, abdomen, and pelvis was obtained. The only positive finding on CT imaging was non-specific right axillary lymph node enlargement. Cytomegalovirus (CMV) and Epstein-Barr virus (EBV) PCR in blood were negative. Serology for EBV and CMV showed past infection.

**Table 2:** Maximum temperature during the hospital course.

Day of admission	Temperature, °F (°C)
Day 1	102.9 (39.3)
Day 2	103.3 (39.6)
Day 3	103.1 (39.5)
Day 4	102.9 (39.3)
Day 5	103 (39.4)
Day 6	102.3 (39)
Day 7	102.9 (39.3)
Day 8	102.8 (39.3)
Day 9	102.1 (38.9)
Day 10	102.1 (38.9)
Day 11	99.3 (37.3)
Day 12	101 (38.3)

Vancomycin was discontinued on day two because methicillin-resistant *Staphylococcus aureus* (MRSA) was not isolated in blood cultures, and piperacillin/tazobactam was discontinued on day five because cultures were negative, and there was no evidence of a gram-negative bacterial infection.

On day five, in view of persistent fever (Table 2), a history of having cats, and borderline axillary lymph nodes, the patient was started empirically on treatment for cat’s scratch disease with azithromycin. This treatment continued for five days and was discontinued as serology was negative, and the patient continued spiking fever despite antibiotics (Table 2). An autoimmune workup was also ordered (Table 3).

**Table 3:** Autoimmune workup findings.

Laboratory parameters	Results	Reference range and units
Anti-DNA antibody	<1.0	<30 IU/mL, negative
Anti-nuclear antibody	1: 40, speckled pattern	1:40, negative
Anti-RNP antibody	<1.0	<1.0 IU/mL, negative
*Anti*-*Smith antibody*	<1.0	<1.0 IU/mL, negative
Anti-Jo antibody	<1.0	<1.0 IU/mL, negative
Rheumatoid factor	<10	≤14 IU/mL, negative
C3 complement	149	90–150 mg/dL
C4 complement	30	16–47 mg/dL
Antibody assay, ribosomal P protein	<1	<1.0 IU/mL, negative
Antibody assay, human T-cell lymphotropic virus types 1 and 2	Non-reactive	
Fluorescent treponemal *antibody*	Reactive	1:4 baseline RPR
Toxoplasma antibody IgG	<7.20 IU/mL	<9 IU/mL, negative
Toxoplasma antibody IgM	<8 IU/mL	8–9.9 IU/mL
*Bartonella henselae* IgM	Negative	
*Bartonella henselae* IgG	Negative	
*Bartonella quintana* IgM	Negative	
*Bartonella quintana* IgG	Negative	

In addition, toxoplasma, *Bartonella*, human T-cell lymphotropic virus types 1 and 2 serologies, and QuantiFERON TB gold were negative.

On day six of admission, the patient expressed some tenderness in the right axillary lymph node. On day seven, the patient underwent a gallium scan which did not reveal any abnormal uptake. Interventional radiology was consulted to perform a biopsy of the tender right lymph node which was performed on day 11 of hospitalization.

One day after the biopsy was taken, the patient was discharged in stable condition and prescribed acetaminophen for fever, with a follow-up with the primary care doctor.

The patient followed up in the office on day 23 after his initial presentation to the Emergency Department. He revealed that his fever broke spontaneously on day 20th. He reported fatigue and swelling in the right axillary region. The patient reported visiting a different emergency room in New York City, where he underwent a repeat CT of the chest which showed a small left pleural effusion. The patient was discharged on the same date as the emergency visit.

**Figure 1:**
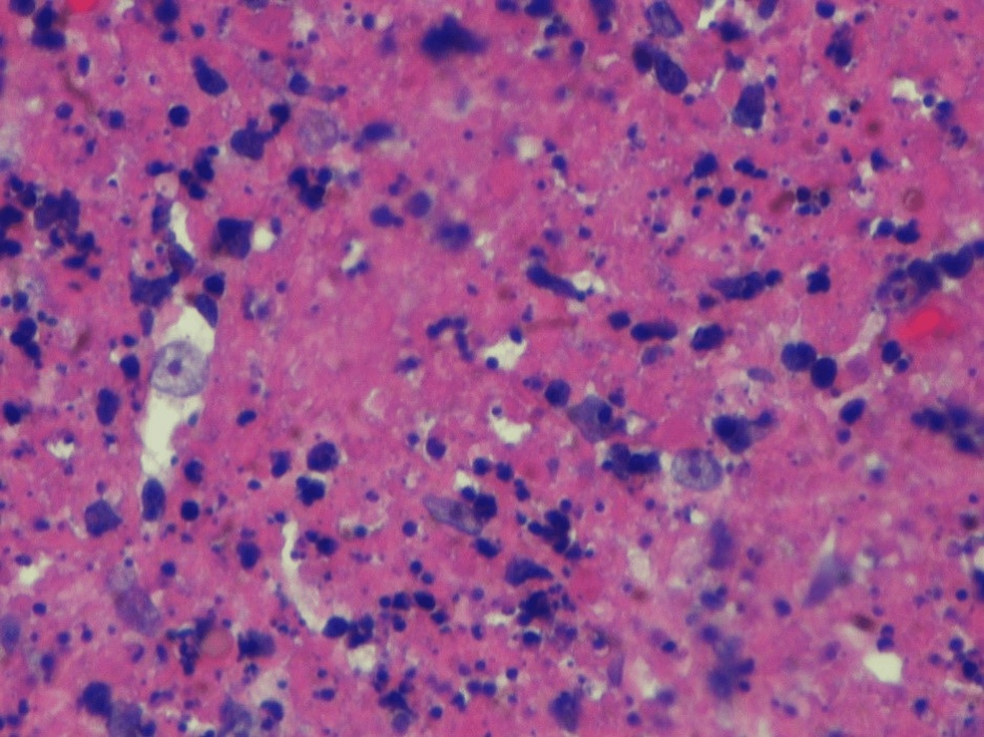
The affected nodes show focal well-circumscribed, paracortical, necrotizing lesions.

**Figure 2:**
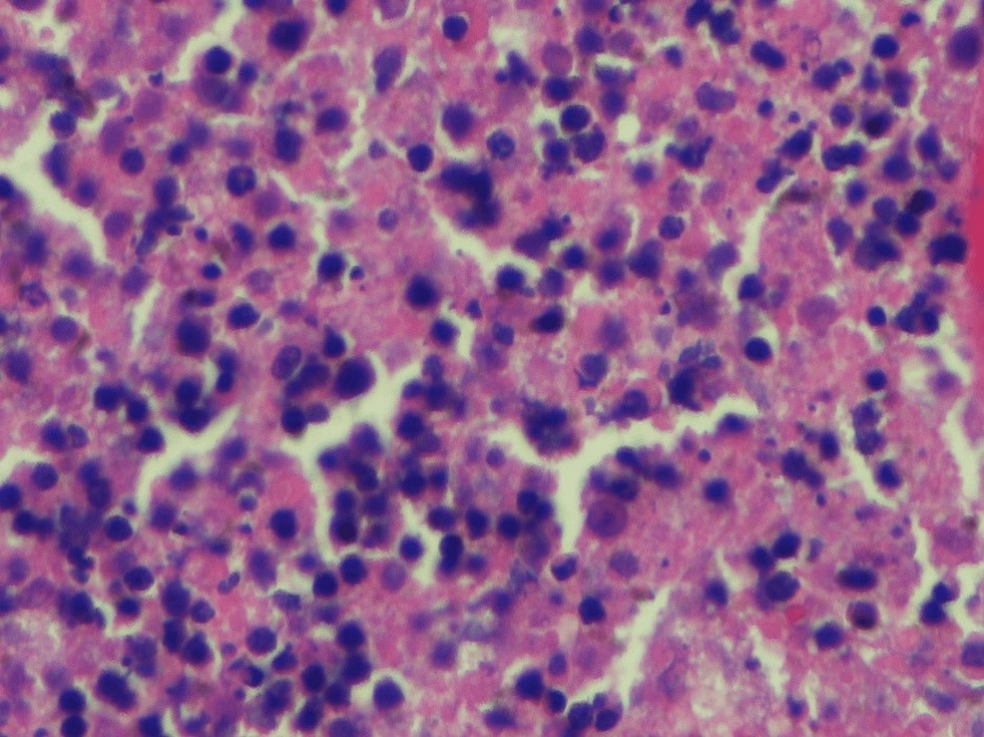
Abundant karyorrhectic debris, scattered fibrin deposits, and collections of large mononuclear cells.

The patient followed up again in the office on day 26 for biopsy results which showed KFD (Figures 1, 2). On day 26, the patient presented complaining of pain with deep inspiration, which was mild but uncomfortable. Troponin T serum was negative. Electrocardiogram did not show findings suggestive of myocarditis.

After a discussion with the patient, he was given 0.5 mg/kg prednisone daily for five days. After 48 hours of steroids, the patient had a resolution of his symptoms of pleuritic chest pain. The patient has been followed for six months after discharge without evidence of autoimmune disease.

## Discussion

KFD, or Kikuchi’s disease, was first described in 1972 by two Japanese pathologists, Kikuchi and Fujimoto [4,5]. Although initially mainly reported in Asia, it is currently reported worldwide [6,7]. The etiology of KFD remains unknown. Many viruses such as EBV, CMV, human herpesvirus 6 and 8, and parvovirus B19 have been implicated as a cause of KFD. However, the etiology of KFD has not been established so far [8-10]. Some studies have also shown the association of KFD with non-infectious conditions such as Still’s disease, systemic lupus erythematosus, mixed connective tissue disease, antiphospholipid syndrome, thyroiditis, polymyositis, scleroderma, and autoimmune hepatitis suggesting an autoimmune etiology [7,11-14].

KFD is a self-limiting disease. The most common presentation is localized lymphadenopathy, although few cases of generalized lymphadenopathy have been reported [15]. Fever is commonly reported in different case series, with frequency ranging from 6.7% to 73.5% [6]. There have been reports of fever of unknown origin caused by KFD or prolonged fever of more than two weeks [16-22]. Our patient also had a similar presentation of prolonged fever. In all reported cases, there was an extensive infectious workup. Our patient had documented fever for 11 days and self-reported persistent fever for 20 days, which was very distressing to the patient and was a cause of the extensive infectious workup. Other non-specific symptoms include night sweats, arthralgia, rash, and hepatosplenomegaly [6]. Our patient only reported malaise and later pleuritic chest pain. In the laboratory, leukopenia, as seen in our patient, and inflammatory syndrome have been reported. Our patient had a high D-dimer but normal C-reactive protein. The rate of recurrence has been estimated to be between 3% and 20.6% [6]. There is no established treatment for KFD [23], and the management is mainly supportive care with antipyretics and analgesics as required.

Steroids have been used to successfully improve symptoms in patients with prolonged fever of more than two weeks, especially when malignant lymphoma and tuberculosis have been ruled out [18]. There have been reports of KFD associated with hemophagocytic syndrome with fatal consequences [24].

Recently, studies have been conducted to understand the role of positron emission tomography scans in identifying KFD and have shown utility in the majority of patients with hypermetabolic lymph nodes [25,26].

The diagnosis of KFD can only be achieved with a biopsy of the lymph node which shows irregular paracortical areas of necrosis and large numbers of different histiocytes at the margin of the necrotic regions [13]. Prompt biopsy should be performed to obtain a rapid diagnosis and avoid extensive workup.

The treatment of KFD is usually symptomatic, including non-steroidal anti-inflammatory drugs and antipyretics. In severe cases, corticosteroids can be used if symptoms do not respond to symptomatic treatment. Other immunosuppressive agents such as hydroxychloroquine, cyclosporine, and azathioprine can also be useful [27].

## Conclusions

KFD is a rare disease that usually affects young females and presents with cervical lymphadenopathy. Establishing an early diagnosis helps in preventing further invasive investigations. Although the disease can be challenging to diagnose, it typically resolves spontaneously. However, KFD cases with severe pulmonary infection and secondary hemophagocytic lymphohistiocytosis may have a fatal course. Regular follow-up with a healthcare provider is crucial for managing KFD and ensures optimal patient outcomes.
